# Photodynamic Eradication of *Trichophyton rubrum* and *Candida albicans*

**DOI:** 10.3390/pathogens10030263

**Published:** 2021-02-25

**Authors:** Anton Valkov, Michael Zinigrad, Marina Nisnevitch

**Affiliations:** Department of Chemical Engineering, Ariel University, Kiryat-ha-Mada, Ariel 4070000, Israel; antonva@ariel.ac.il (A.V.); zinigradm@ariel.ac.il (M.Z.)

**Keywords:** photodynamic treatment, rose Bengal, malachite green, methylene blue, *Trichophyton rubrum*, *Candida albicans*

## Abstract

Conventional methods of onychomycosis treatment are ineffective in some cases because the cure of onychomycosis very often depends on the patient’s individual response to the treatment; therefore, there is a crucial need to research and develop new methods of onychomycosis therapy. One of the most innovative treatments is photodynamic therapy (PDT) using photosensitizers (PSs). However, effective treatment depends on the correct choice of photosensitizer and substances that improve the characteristics of the final formulation. The aim of our work was to find an effective formulation for the treatment of onychomycosis. To achieve this goal, we tested the effect of three types of PSs, rose Bengal (RB), malachite green oxalate (MGO), and methylene blue (MB), on *Candida albicans*. The most effective PS was RB, and so the study was continued with *Trichophyton rubrum*. Additional comparative studies were carried out on substances included in the formulation (urea and thiourea), focusing on their antifungal activity, which can improve penetration through the nail plate. The composition of the formulation that achieved 100% eradication of *Trichophyton rubrum* under our conditions consisted of 150 μM RB, 5% urea, and 0.5% thiourea in glycerol/water (70/30%, *w*/*w*) solution. A white luminescent lamp was used as a light source (1.9 ± 0.1 mW cm^−2^). Stability of the formulation was checked. The selected formulation shows potential for future simplification and acceleration of PDT treatment of onychomycosis.

## 1. Introduction

Onychomycosis, fungal nail infection, is an acute and persistent problem in public health. Epidemiological studies around the world have found a high incidence of onychomycosis in at least 10–30% of the general population [1,2], in more than 20% of those over 60 years old, in 50% of those over 70 years old, and in 30% of patients with diabetes [3,4].

Onychomycosis is often considered only an aesthetic problem. However, patients having onychomycosis along with a background of immunodeficiency or endocrine diseases may develop widespread mycosis of the skin. Sometimes onychomycosis is accompanied by development of severe complications such as diabetic foot, extremity erysipelas, or lymphostasis. For this reason, onychomycosis treatment is strictly necessary and should be carried out promptly [5,6,7]. Clinical studies have shown that the effectiveness of systemic antimycotics after the end of treatment is 40–80%, and after 5 years only 14–50% [8]. It was proven that the effectiveness of onychomycosis therapy can be increased by applying complex methods of treatment that combine antifungal drugs with agents affecting pathogenesis [9,10].

Advanced methods of onychomycosis therapy include local or oral therapy and surgical intervention [1,11]. However, even when using these complex methods, it is difficult to achieve positive results, since drugs applied topically usually cannot penetrate into the nail plate to the site of infection, but instead are concentrated on its external surface [12]. Moreover, systemic use of antifungal drugs such as ketoconazole, terbinafine, and griseofulvin sometimes needs up to 12 months of treatment [13], and long-term application of these drugs carries a toxicological effect that can harm human health [14,15]. In addition, fungi may develop resistance to antifungal drugs under a long period of exposure. Surgical treatment is very unpopular, since it causes discomfort and pain in patients and is relatively ineffective since it leads to deformation of the treated nail plates and scar formation [16,17].

In recent years, alternate methods of onychomycosis treatment, such as photodynamic therapy (PDT), have been actively developed and tested [18]. PDT is a special field of light therapy. Compounds called photosensitizers (PSs) are applied to an infected site and undergo activation by visible light [19,20]. Illumination leads to production of active radicals, such as reactive oxygen species (ROS), which cause irreversible damage to microorganisms. The ability of PSs to eradicate microorganisms has been described by many authors [21,22,23,24,25,26,27,28]. PSs are effective against bacterial, viral, fungal, and protozoal infections [29].

To enhance the efficiency of PDT, it is necessary to select the PS formulation with optimal consistency in providing high-speed drug penetration into a nail plate. However, although necessary, this parameter is not a sufficient condition for successful local treatment of fungal infections. To assess the effectiveness of onychomycosis treatment with antifungal drugs in various forms (e.g., transfersomes, nanoemulsions, gels, creams, and ointments) and their combinations, with or without the addition of surfactants [30,31], the use of keratin biomembranes was proposed as a good substitute for hard-to-obtain human nails [32,33,34]. The keratin biomembranes have physical properties similar to nail plates and can be obtained with good reproducibility.

This study aimed to find a promising formulation and conditions for effective PDT against fungal infection caused by *Candida albicans* and *Trichophyton rubrum*. Three water-soluble PSs were selected for antifungal studies, rose Bengal (RB), malachite green oxalate (MGO), and methylene blue (MB), all of which exhibit high antimicrobial activity [35,36]. These PSs were tested against fungi at different concentrations and in various formulations, with and without keratolytic agents. 

An appropriate formulation for photodynamic treatment of onychomycosis should include a photosensitizer and keratolytic agents to increase the permeability of nail plates to active agents. The formulation should also have a viscosity high enough to keep the PS on the nail surface and prevent its leakage to the nail sides. We also examined the stability of the proposed formulations. 

## 2. Results and Discussion

This work focused on a study of antifungal properties of photosensitizers, alone and in combination with factors affecting the permeation of drug molecules into a nail plate. Two fungi known to cause onychomycosis were examined: *Candida albicans* and *Trichophyton rubrum.* The former strain was tested in planktonic cultures and the latter on solid media including fabricated keratin membranes that model nail plates.

### 2.1. Photodynamic Treatment of Planktonic Culture of Candida albicans 

**Figure 1:**
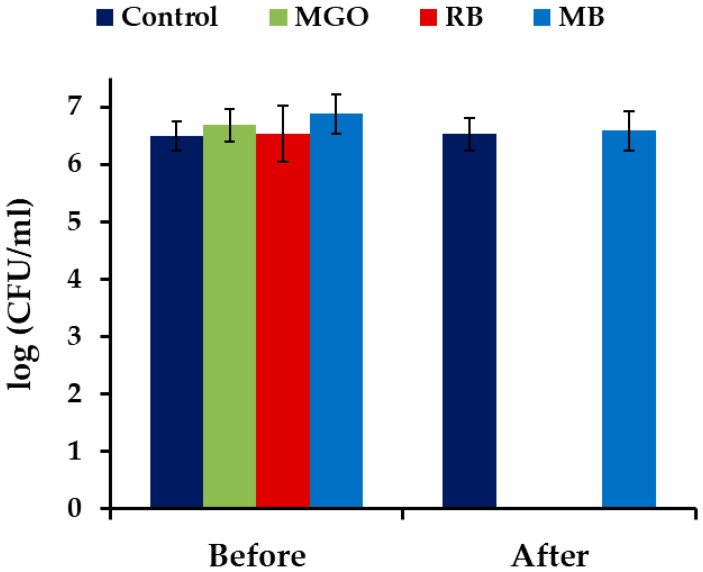
The effect of malachite green oxalate (MGO), rose Bengal (RB) and methylene blue (MB) at 500 μM concentration on planktonic cultures of *Candida albicans* under white-light illumination at 1.9 ± 0.1 mW/cm^2^ fluence for 4 h.

First, the chosen PSs (RB, MGO, and MB) were tested on planktonic cultures of *Candida albicans* using a high concentration (500 µM) of each PS under prolonged illumination. For this purpose, the solutions were distributed into Petri dishes containing *Candida albicans* suspension and illuminated for 4 h by a white luminescent light. The experiment showed that only MGO and RB caused total eradication of *Candida albicans* cells, while MB was inactive (Figure 1). In our previous studies, MB showed high photodynamic activity against Gram-positive and Gram-negative bacteria [22,35,37,38,39], however, in the case of fungi, this PS was unable to inhibit cell growth.

**Figure 2:**
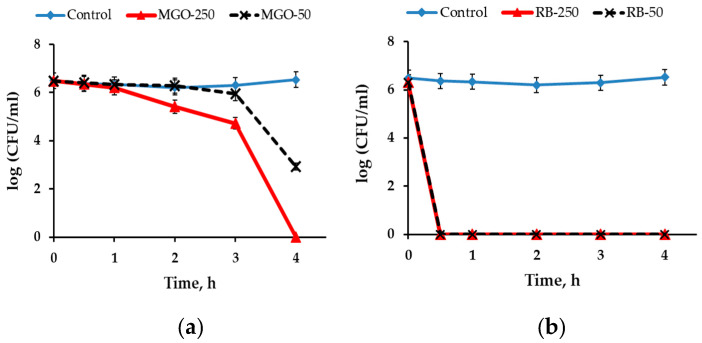
Effect of 50 and 250 μM of (**a**) MGO and (**b**) RB on planktonic culture of *Candida albicans* under white-light illumination at the fluence of 1.9 ± 0.1 mW/cm^2^.

Since only two PSs showed antifungal activity, the study was continued using MGO and RB only. At the next stage, their photodynamic activity was tested at different concentrations and during various time periods. The effect of MGO on *Candida albicans* is shown in Figure 2a. Complete inactivation of *Candida albicans* was achieved using MGO at 250 μM concentration after 4 h of illumination, compared to 50 μM MGO, which caused only partial cell destruction. Over shorter time periods, even 250 μM MGO decreased the cell concentration only by 0.5–2 log_10_ values. The activity of RB against *Candida albicans* is illustrated in Figure 2b. The cells were completely eradicated at concentrations of both 50 and 250 μM RB after 30 min of illumination, i.e., RB showed a significantly higher ability in fungal cell eradication than did MGO. 

**Figure 3:**
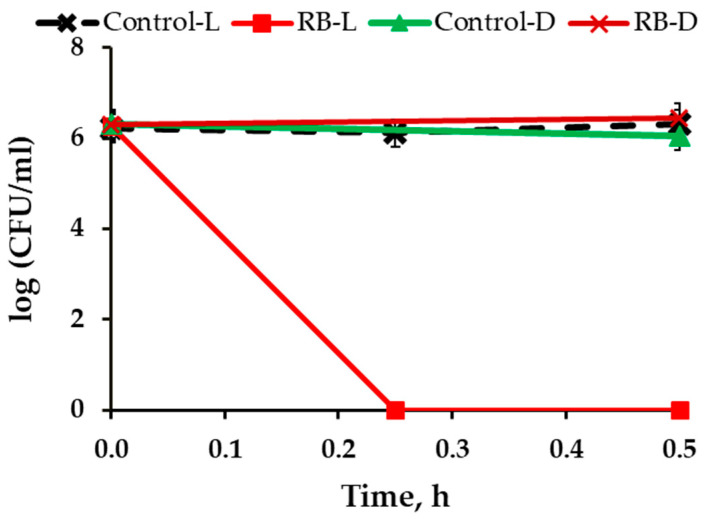
Treatment of *Candida albicans* planktonic culture by 50 μM RB under white-light illumination at the fluence of 1.9 ± 0.1 mW/cm^−2^ (L) and in the dark (D).

Next, it was proven that the cells were eradicated by RB due to illumination. For this purpose, the photodynamic activity of RB against *Candida albicans* was compared to the same process in darkness. The results in Figure 3 show that under illumination, 50 μM RB totally eradicated *Candida albicans* within 15 min, whereas in the dark, even after 30 min the fungi cell concentration did not differ from that in the control series. Previously, the photodynamic effect of RB on planktonic cultures of *Candida albicans* was demonstrated by Costa et al. [40] and Freire et al. [41]. Costa et al. achieved a 1.58 log_10_ reduction in cell concentration under treatment with 5 μM RB, using a blue LED light at 455 ± 20 nm at the fluence rate of 526 mW/cm^2^ and illumination for 180 s, which supplied an energy dose of 95 J/cm^2^. Freire et al. used 12.5 μM RB under a green LED light at 532 ± 10 nm at a fluence rate of 237 mW/cm^2^ and the same illumination time (energy dose 42.6 J/cm^2^), thereby achieving complete cell eradication. In our study, complete cell eradication was obtained at a higher RB concentration (50 μM) but at much lower light fluence (2.0 mW/cm^2^). In our experiments, using 50 μM RB illuminated by a white wide-spectrum lamp supplying an energy dose of only 1.7 J/cm^2^ enabled the complete inhibition of *Candida albicans* growth.

### 2.2. Photodynamic Treatment of Trichophyton rubrum

**Figure 4:**
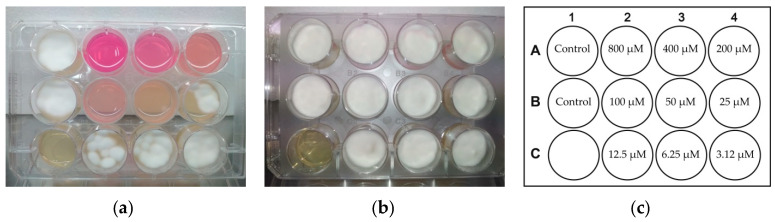
Effect of aqueous solutions of RB at various concentrations on growth of *Trichophyton rubrum* on Yeast Mold (YM) agar: (**a**) under illumination of 1.9 ± 0.1 mW/cm^2^ for 30 min and (**b**) in the dark. The layout of wells in the experiment (**c**): control wells A1 and B1—untreated culture of *Trichophyton rubrum*; control C1—agar medium without fungal culture; the remaining wells—agar medium with applied aqueous solutions of RB at concentrations specified in the scheme.

**Figure 5:**
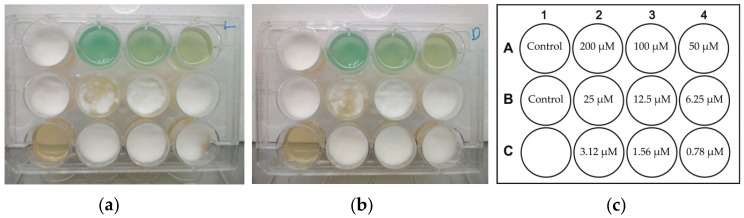
Effect of aqueous solutions of MGO at various concentrations on growth of *Trichophyton rubrum* on YM agar: (**a**) under illumination of 1.9 ± 0.1 mW/cm^2^ for 30 min and (**b**) in the dark. The layout of wells in the experiment (**c**): control wells A1 and B1—untreated culture of *Trichophyton rubrum*; control C1—agar medium without fungal culture; the remaining wells—agar medium with applied aqueous solutions of MGO at concentrations specified in the scheme.

In this part of the study, we tested two prospective photosensitizers, MGO and RB, against the fungus *Trichophyton rubrum*. In addition, we examined the antifungal effect of two common keratolytic agents, urea and thiourea [42]. Solutions of the agents were prepared in a water base or a glycerol/water (70/30%, *w/w*) mixture. First, we studied the photodynamic effects of RB and MGO in aqueous solutions at various concentrations (Figure 4 and Figure 5, respectively). Figure 4a,b show the effect of RB on the cells of *Trichophyton rubrum* under illumination and in the dark, respectively. The layout of wells in the experiment is shown in Figure 4c. Complete inhibition of *Trichophyton rubrum* growth was achieved in the presence of 50 μM RB under illumination (Figure 4a). RB had no effect on *Trichophyton rubrum* in the dark. 

It is worth mentioning that the cells of *Candida albicans* were also inhibited by RB at this concentration (Figure 2b). In the work of Houang et al. [43], it was shown that RB at the concentration of 140 μM can kill virtually all (99.99 ± 0.01%) spores of *Trichophyton rubrum* under irradiation by a 532 nm laser at 40 mW for 10 min (101 J/cm^2^ fluence). In clinical studies, RB was applied at the concentration of 140 μM under laser irradiation at 2.55 W/cm^2^ against toenail onychomycosis in vivo and after 5 treatments for 3 months complete clinical and mycological clearance was achieved [44].

A similar experiment was performed using MGO, and the results showed that this PS also totally inhibited the growth of *Trichophyton rubrum* at 50 μM, but unlike RB, there was no difference between the results obtained under illumination and in the dark (Figure 5a,b, respectively). Figure 5c presents the layout of wells in this experiment. Since the effect of MGO on fungal cells could not be assigned to its photodynamic activity, the study was continued using only RB as a PS.

**Figure 6:**
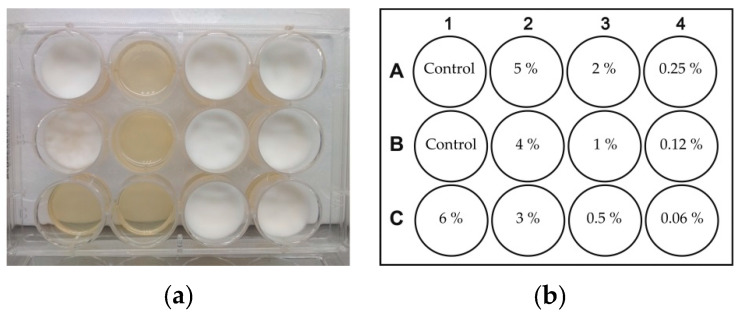
Effect of thiourea at various concentrations on growth of *Trichophyton rubrum* on YM agar medium (**a**). A layout of wells (**b**) containing aqueous solutions of thiourea applied to agar medium at concentrations specified in the scheme (%, *w/v*). Control wells contained no thiourea. The experiment was performed under illumination of 1.9 ± 0.1 mW cm^−2^ for 30 min.

**Figure 7:**
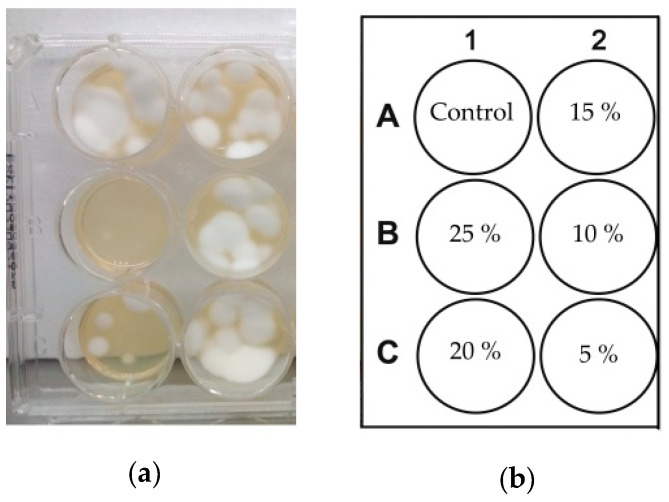
Effect of urea at various concentrations on growth of *Trichophyton rubrum* on YM agar medium (**a**). A layout of wells (**b**) containing aqueous solutions of thiourea applied to agar medium at concentrations specified in the scheme (%, *w/v*). Control wells contained no urea. The experiment was performed under illumination of 1.9 ± 0.1 mW/cm^2^ for 30 min.

Two agents known to enhance the permeability of nails, urea and thiourea [42,45], were tested for antifungal activity as well. The experiments were performed as in the case of the PSs. Figure 6 and Figure 7 show the antifungal effects of thiourea and urea, respectively, when applied in aqueous solutions to agar medium with pre-seeded fungi.

The results showed that thiourea exhibited strong antifungal effects at concentrations above 3% (Figure 6), while urea totally inhibited the growth of *Trichophyton rubrum* at concentrations of above 25% (Figure 7). Previous in vivo studies showed contradictory data on the antifungal effect of urea. Bunyaratavej at al. [46] reported a partial (32%) cure of onychomycosis when using a cream containing 40% urea. However, Escalante et al. [47] found that application of the same 40% urea cream led to an onychomycosis mycological cure in 8.3% of cases.

**Figure 8:**
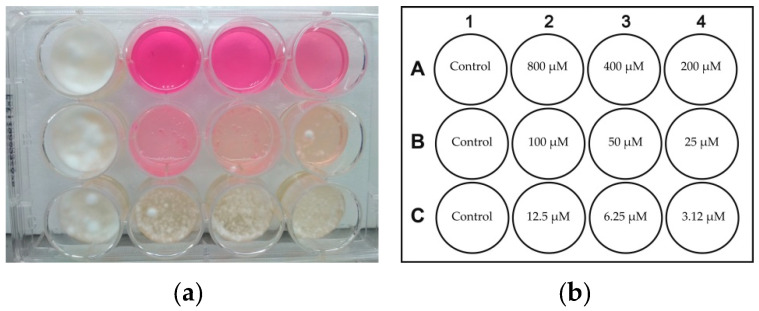
Effect of glycerol/water (70/30%, *w/w*) solutions of RB at various concentrations on growth of *Trichophyton rubrum* on YM agar, (**a**) under illumination of 1.9 ± 0.1 mW/cm^2^ for 30 min. A layout of wells (**b**): control wells A1, B1, and C1—untreated culture of *Trichophyton rubrum*; the remainder—agar medium with applied glycerol/water solutions of RB at concentrations specified in the scheme.

At the next stage of our study, we tested the antifungal activity of RB, a mixture of urea and thiourea, and a mixture of RB, urea and thiourea in a glycerol/water solution. Figure 8 shows the effect of RB in a glycerol/water mixture on *Trichophyton rubrum*. Fungal growth was totally inhibited at RB concentrations above 200 μM. Although it is not seen distinctly in the photograph in Figure 8a, at concentrations of 50 and 100 μM there was a proliferation of fungal cells. 

It should be mentioned that this inhibitory concentration of 200 μM is much higher than that of RB in an aqueous solution (50 μM). We assume that such a difference between these results may be due to the different amounts of dissolved oxygen in the solutions. Oxygen solubility decreases linearly with an increase of glycerol fraction in glycerol/water mixtures [48], so the oxygen concentration may be insufficient for providing effective photodynamic activity of PSs acting according to the Type II mechanism. This mechanism involves energy transfer from a PS in a triplet excited state (^3^PS*) to dissolved molecular oxygen in a triplet ground state (^3^O_2_), leading to a formation of oxygen in a singlet excited state (^1^O_2_), which causes irreversible damage to microorganisms [49,50].

**Figure 9:**
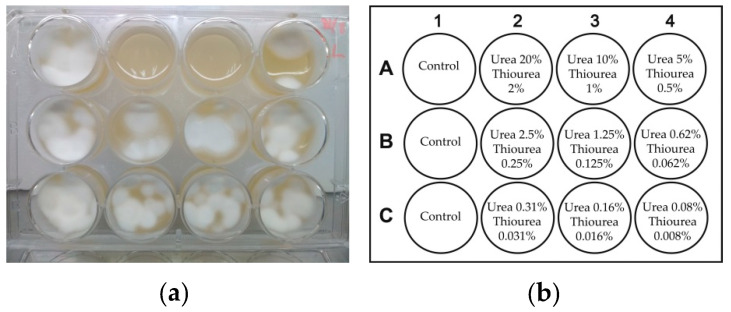
Effect of urea/thiourea in various concentrations on growth of *Trichophyton rubrum* on YM agar medium (**a**). Layout of wells (**b**) containing glycerol/water (70/30%, *w/w*) solutions of urea/thiourea applied onto agar medium at concentrations specified in the scheme (%, *w/v*). Control wells contained no urea/thiourea. The experiment was performed under illumination of 1.9 ± 0.1 mW/cm^2^ for 30 min.

After determining the concentrations of thiourea and urea in aqueous solution that inhibit growth of *Trichophyton rubrum*, the antifungal effects of their mixture in a glycerol/water solution was tested. Since urea and thiourea inhibited *Trichophyton rubrum* at concentrations of 25% and 3%, respectively, which is an approximate ratio of 10:1, this ratio was chosen as a baseline for all dilutions in this experiment. The results are presented in Figure 9. Interestingly, upon mixing urea and thiourea, the inhibiting of fungal growth occurred at much lower concentrations of these components than when applying each of them separately. The same inhibitory effects on *Trichophyton rubrum* obtained at the above-mentioned concentrations of urea and thiourea when applied separately (25% and 3%, respectively) was achieved by their mixture at concentrations of only 10% and 1%, respectively (Figure 9). The results obtained in the dark were identical to those obtained under illumination (data not shown). It appears that the mixture caused a synergistic antifungal effect despite the addition of glycerol to the solution. 

**Figure 10:**
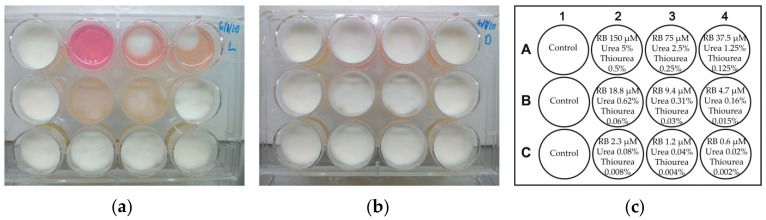
Effect of RB, urea, and thiourea at various concentrations in glycerol/water (70/30%, *w/w*) solutions on the growth of *Trichophyton rubrum* on YM agar: (**a**) under illumination at 1.9 ± 0.1 mW/cm^2^ for 30 min and (**b**) in the dark. A layout of wells (**c**): control wells A1, B1, and C1—untreated culture of *Trichophyton rubrum*; the remainder—agar medium with applied glycerol/water solutions of RB, urea, and thiourea at concentrations specified in the scheme.

Finally, we tested a formulation containing all three successful compounds (RB, urea, and thiourea) in glycerol/water (70/30%, *w/w*) solutions at various concentrations, with *Trichophyton rubrum,* under illumination and in the dark. Figure 10 shows the results of this experiment. Under illumination, total inhibition of fungal growth was observed in the formulation containing 150 μM RB, 5% urea, and 0.5% thiourea (Figure 10a). Figure 10b shows that without illumination the formulation rendered no antifungal effect. The mixture of urea and thiourea at the concentrations applied in this experiment did not inhibit *Trichophyton rubrum,* and the inhibiting concentration of RB in this formulation was lower than when RB was applied alone. Therefore, the presence of urea and thiourea even in sub-inhibiting concentrations contributed to the overall antifungal effect.

### 2.3. Stability of Formulations

**Figure 11:**
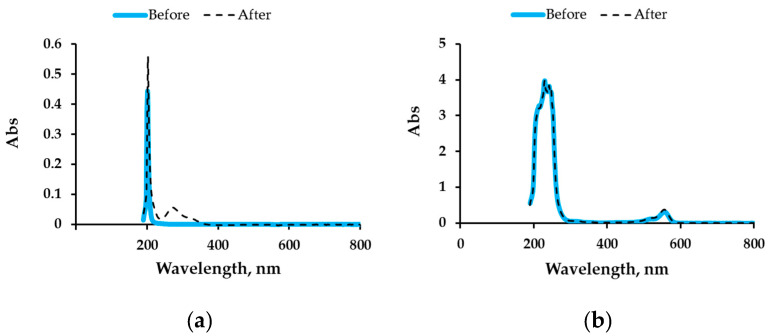
UV-visible absorption spectra of the urea solution (**a**) and the formulation containing RB, urea, and thiourea (**b**) in a glycerol/water (70/30%, *w/w*) solution before (blue line) and after 32 days of incubation (black dotted line) at 30 °C in the dark.

To examine the stability of the selected antifungal formulation over time, eight solutions, containing each component alone or a mixture of all three components in aqueous and glycerol/water solutions (Table 1), were prepared at the same concentrations of stock solutions used for the photodynamic experiments described above. The solutions were incubated under shaking in the dark and monitored by measuring their UV/Vis spectra over time. The monitoring of the solutions was performed at wavelengths of maximal absorption, λmax (Table 1). With regard to the four water-based and three glycerol/water solutions (samples 1–4 and 6–8 in Table 1, respectively), no changes in their spectra were observed during 32 days of monitoring; therefore, it was concluded that these samples were stable over time. However, the spectrum of the urea solution in the glycerol/water mixture (sample 5 in Table 1) changed after the 32-day incubation, displaying a new peak at 272 nm (Figure 11a), probably due to decomposition of the urea. Since the antifungal formulation suggested as optimal contains all three compounds (urea, thiourea, and RB) in the glycerol/water mixture, we assume that urea may decompose in this formulation as well. Nevertheless, its spectra (sample 8, Figure 11b) did not register any additional peak, or even a shoulder, in the region of 250–300 nm. Therefore, we concluded that this formulation demonstrates good overall stability.

We assume that in vivo experiments will show the suggested formulation to be efficient against onychomycosis. Moreover, using an ordinary white luminescent lamp as proposed here contrasts with most studies on photodynamic inhibition of fungi, where excitation of PSs is performed by lasers at specific wavelengths. This difference should simplify future applications of our suggested formulation for onychomycosis treatment in everyday environments. 

To complete the determination of optimal conditions of the treatment, our group is currently carrying out studies on PS permeation into keratin biomembranes that model nail plates. These studies are attempting to test the effect of the keratolytic agents (urea and thiourea) on delivery of PSs into the nail plate.

## 3. Materials and Methods

### 3.1. Fungal Strains

Two fungal strains *Candida albicans* (ATCC 90028) and *Trichophyton rubrum* (ATCC MYA-4438) were used in this study.

### 3.2. Preparation and Growth of Fungi 

*Candida albicans* was seeded onto Difco^TM^ Yeast Mold (YM) agar (BD, Franklin Lakes, NJ, USA) and incubated for 48 h at 30 °C; 2–3 colonies of yeast were seeded into Difco^TM^ YM broth (BD) and incubated under shaking at 200 rpm for 24 h at 30°C. The obtained inoculum was diluted with saline (0.90% *w/v* of NaCl) to obtain a suspension having OD_530_ = 0.10–0.16. This OD was measured using a spectrophotometer (Genesys 10S UV-VIS, Thermo Fisher, Waltham, MA, USA), and its value corresponded to cell concentration of 1–3 × 10^6^ cells/ml according to the 0.5 McFarland standard method [51].

*Trichophyton rubrum* was grown for 14 days on YM agar at 30 °C. Samples of 0.5 cm^2^ of dermatophyte culture were cut off from the agar plates with a scalpel, suspended in 10 mL of YM broth and incubated under shaking at 200 rpm for 48 h at 30 °C. The obtained suspensions were filtered through a sterile cotton gauze pad, which retained hyphal fragments but permitted the passage of dermatophyte microconidia.

### 3.3. Photodynamic Treatment of Planktonic Cultures of Candida albicans 

Stock solutions of RB, MGO, and MB hydrate (Sigma-Aldrich, St. Louis, MO, USA) in water were prepared at the concentration of 4.5 mM. Aliquots of the stock solutions were added to 10 mL of *Candida albicans* suspension at the concentration of 2–8 × 10^6^ cells/mL in a Petri dish, to achieve a final PS concentration of 500 μM. In some experiments, RB and MGO solutions were added to 10 mL of *Candida albicans* suspension to obtain final concentrations of 50, 250, or 500 μM. The cell suspensions with MB were exposed to light for 4 h, and samples with RB and MGO were exposed for 0.5, 1, 2, 3, and 4 h, using an 18 W white luminescent lamp with an emissions range of 400–700 nm, providing a light intensity of 1.9 ± 0.1 mW/cm^2^ at ambient temperature. Illuminance was measured by a LX-102 light meter (Lutron, Taipei, Taiwan). After irradiation, 100 μL aliquots of each sample in 3–4 decimal dilutions were spread over YM agar plates with a Drigalsky spreader, incubated at 37 °C overnight, and then the colony-forming units (CFU) were counted. These experiments aimed to test the photodynamic character of the PS activity compared to activity without exposure to light. In the control experiments, samples of *Candida albicans* suspension without addition of PS were used.

### 3.4. Photodynamic Treatment of Trichophyton rubrum 

**Scheme 1:**
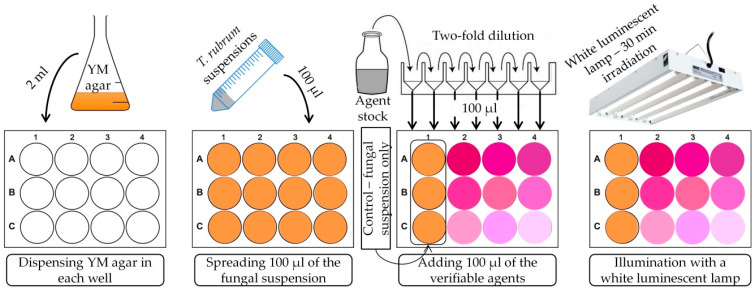
General presentation of the experiment on the photodynamic treatment of *Trichophyton rubrum*.

The schematic presentation of experiments in the photodynamic treatment of *Trichophyton rubrum* is shown in Scheme 1. First, 2 mL portions of YM agar were dispensed into each well of a 12-well plate. After hardening of the agar medium, 100 µL aliquots of *Trichophyton rubrum* suspension were added on the surface. After that, 100 µL of PS solutions, in water or in a glycerol/water (70/30%, *w/w*) mixture, were applied to the surface after two-fold dilutions. In the case of water solutions, the final concentration in those added onto the agar surface ranged between 800 to 3.12 µM for RB, 200 to 0.78 µM for MGO, 25% to 5% (*w/v*) for urea, and 7% to 0.06% (*w/v*) for thiourea. In the case of glycerol/water solutions, the final concentration on the agar surface ranged between 800 to 3.12 µM for RB, and 20/2% to 0.078/0.008% (*w/v*) for urea/thiourea. The concentration in the three-component formulation (RB/urea/thiourea) was between 150 µM/5%/0.5% and 0.6 µM/0.02%/0.002% (*w/v*), respectively. 

After addition of *Trichophyton rubrum* suspensions and the solutions of components specified above, the 12-well plate was illuminated for 30 min with a white luminescent lamp at the light fluence of 1.9 ± 0.1 mW/cm^2^. All experiments were performed at ambient temperature. After illumination, the plates were incubated in the dark at 30 °C for 7 days and examined visually for fungal growth. The same experiments were carried out in parallel without light exposure, and all experiments were duplicated.

### 3.5. Stability of Formulations 

Two solvents were used to prepare eight solutions; four of them were water-based (samples 1–4) and four were based on a glycerol/water (70/30%, *w/w*) mixture (samples 5–8). Table 1 presents the prepared solutions, containing 0.1 g of each compound (urea, thiourea, or RB, and the three compounds together) dissolved in 10 mL of each solvent. All the solutions were incubated under shaking at 200 rpm for 32 days at 30 °C in the dark. Aliquots of 100 µL from each solution were sampled at the beginning of the experiment, and after 4, 7, and 32 days of incubation. Before examination, the samples were diluted with water, and their spectra were then registered using a 1 cm quartz cuvette in the range of 190–1100 nm with steps of 2 nm. Further monitoring of the solutions was performed at wavelengths of maximal absorption λmax (Table 1).

### 3.6. Statistical Methods

Each experiment was carried out in duplicate and then repeated to confirm the reproducibility of results. The average value ± SD was calculated.

## 4. Conclusions

Three photosensitizers, RB, MB, and MGO, were tested for antifungal activity, but only RB was active against *Trichophyton rubrum* and *Candida albicans* under illumination. To find a formulation suitable for onychomycosis treatment, urea and thiourea were added to RB as factors for potential enhancement of nail plate permeability. The most effective formulation for inhibiting fungal growth contained 150 μM RB, 5% urea, and 0.5% thiourea in a glycerol/water (70/30%, *w/w*) solution. The formulation was stable for at least one month of storage at 30 °C. 

## Figures and Tables

**Table 1:** Composition and wavelengths of maximal absorption (λmax) of samples in experiments for testing stability of formulations.

Solvent	Sample Number	Urea (g)	Thiourea (g)	Rose Bengal (g)	λmax (nm)
Water ^1^	1	0.1			190
	2		0.1		196, 236
	3			0.1	212, 546
	4	0.1	0.1	0.1	230, 546
Glycerol/Water ^1^ 70/30%, *w*/*w*	5	0.1			202
	6		0.1		230
	7			0.1	214, 556
	8	0.1	0.1	0.1	230, 556

^1^ Components were dissolved in 10 mL of the solvent.

## Data Availability

Data is contained within the article.
